# On the role of pore constrictions in gas diffusion electrodes†

**DOI:** 10.1039/d2cc02844a

**Published:** 2022-07-13

**Authors:** Michele Bozzetti, Anne Berger, Robin Girod, Yen-Chun Chen, Felix N. Büchi, Hubert A. Gasteiger, Vasiliki Tileli

**Affiliations:** Institute of Materials, Ecole Polytechnique Federale de Lausanne, Station 12 1015 Lausanne Switzerland vasiliki.tileli@epfl.ch; Chair of Technical Electrochemistry, Department of Chemistry and Catalysis Research Center, Technische Universität München Germany; Electrochemistry Laboratory, Paul Scherrer Institute 5232 Villigen PSI Switzerland

## Abstract

Water management by gas diffusion electrodes is a fundamental aspect of the performance of electrochemical cells. Herein, we introduce the characteristic constrictions size as a descriptor for microporous layers (MPL). This parameter is calculated by volumetric analysis of focused ion beam nanotomography and compared to mercury intrusion porosimetry measurements.

## Abstract 2

A descriptor for the characteristic size of constrictions is introduced to evaluate the water saturation properties of gas diffusion electrodes.

## Graphical abstract

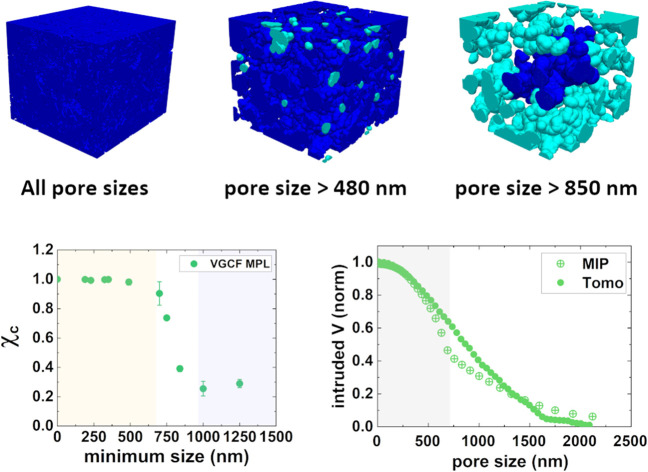

Gas diffusion electrodes play a key role in the mass transport limitations of gas flow in many electrochemical devices including fuel cells^1^ and CO_2_ reduction reaction cells.^2^ In the case of proton exchange membrane (PEM) fuel cells, this mass transport limitation is associated with the liquid water saturation at the cathode.^3^ Typically, gas diffusion layers (GDLs) in PEM fuel cells consist of a dual layer, where a microporous layer (MPL) is added onto a macroporous gas diffusion layer substrate (GDL-S) to enhance the water management during operation.^1^ This effect has been extensively studied using one- and two-phase flows in such complex porous media based on pore network models^4^ and implementing lattice Boltzmann methods^5^ or microstructure–property relationships.^6,7^ In particular, water condensation on the cathode and its impact on the performance of the cells are usually described by traditional GDL attributes that include the tortuosity *τ* and porosity *ε*, which are related through the effective diffusion coefficient^8^ of the gaseous species in the porous medium:1
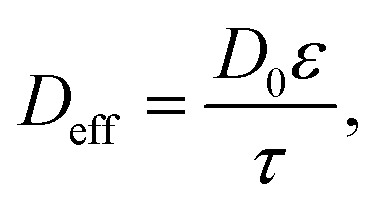
where *D*_0_ is the bulk diffusion coefficient. Another useful descriptor is the characteristic pore size diameter *d*_C_, which can be used to estimate the Knudsen diffusion coefficient, *D*_K_, according to the equation^9^
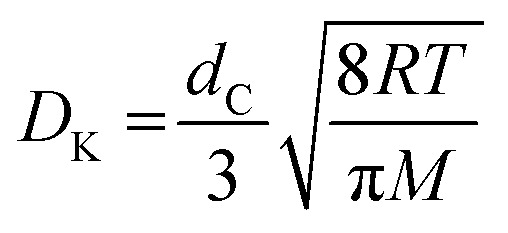
, where *R* is the gas constant, *T* is the temperature, and *M* is the molar mass of the diffusing species. In addition to Knudsen diffusion, *d*_C_ is used in the Washburn equation, which describes water intrusion into hydrophobic and hydrophilic channels,^10^ and in the recently developed pore network models, which relate the local flow rate *q* to the fourth power of the channel size *r* (*q* ∝ *r*^4^).^11^

Many methods have been proposed to experimentally investigate the behaviour of liquid water in the gas diffusion media of PEM fuel cells including X-ray computed tomography (X-CT) operando studies^12^ and neutron imaging.^13^ For example, Shrestha *et al.* showed that preferential paths of liquid water are created along the naturally hydrophilic fibers during operation.^14^ However, the exact hydrophobicity/hydrophilicity at the scale of the MPL pores remains challenging to map. Furthermore, these methodologies cannot achieve the sub-μm scale resolution that reflects the actual size scale of the pores.^15^ Additionally, it has been suggested that larger size pores can better remove liquid water from the catalyst layer,^16^ assuming that larger pore space is connected without constrictions. Therefore, the connectivity of large pore space and existence of narrower constrictions can provide valuable insights concerning the MPL water saturation. Previously, the constrictivity factor *β*^17^ and the constrictivity *δ*^18^ were used as empirical parameters that relate to the effective diffusion coefficient.^19^ Herein, we introduce a different approach: the most representative constriction size of the pore network is calculated and it is used as an MPL descriptor with a direct physical meaning. We utilise nanotomography using focused ion beam/scanning electron microscopy (FIB/SEM),^20–22^ which is a suitable technique to reconstruct three-dimensional (3D) volumes of GDLs and its resolution makes FIB/SEM ideal for acquiring information on the size range of pores found in MPLs.^23^ We implement a novel approach to analyze the 3D tomographic volumes of MPLs by looking at the connected pore volume using the size of pores as a parameter in order to determine the role of the constrictions. For this purpose, two different MPL designs were evaluated: one based on a traditional carbon black network named Li100 and one based on vapor grown carbon fibers (VGCF). These two are known to perform differently under high relative humidity (RH) operating conditions,^16,24^ as it is shown in Fig. S1 (ESI†), and therefore they are ideal to look for new sub-μm scale descriptors.

To reduce surface variation-induced imaging artefacts during FIB/SEM acquisition that could alter the porosity values near the surface, Ar^+^ beam polished cross-sections of the two inherently different MPLs were prepared. Fig. 1a and b show the unembedded cross sections of the Li100 and VGCF MPLs, respectively, deposited onto a Freudenberg H14 (PTFE treated) GDL-S. The thicknesses of the two MPLs were 50 μm for the Li100 and 160 μm for the VGCF based MPLs. Additionally, higher magnification images depicted in Fig. 1c and d reveal that the VGCF MPL texture is less homogeneous than the Li100, displaying larger pores and larger structural features with irregular shapes. Fig. 1c and d show the reconstructed FIB/SEM volumes of Li100 and VCGF MPLs, respectively. The locations of the volume acquisition for the Li100 and the VGCF electrode were taken from the inner parts of the MPLs in order to obtain representative statistics for the bulk phase of the MPLs. The size of the Li100 volume was 760 μm^3^, while for the VGCF an increased volume size of 1311 μm^3^ was acquired, due to its morphology and larger pore size. For both MPL designs the representativity of the volume size was tested by splitting the parent volumes and observing negligible variations between sub-volumes (Fig. S2, ESI†). Further, we calculated the average porosity of Li100 to be *ε*_Li100_ = 0.81 and its tortuosity to be *τ*_Li100_ = 1.22, while for VGCF the porosity was *ε*_VGCF_ = 0.84 and the tortuosity value was *τ*_VGCF_ = 1.16. In both cases, tortuosity was measured in the out-of-plane direction of the MPLs by flow-based simulations, and its values, plotted in Fig. 1e, are in good agreement with most of the tortuosity calculations at such porosity values.^25,26^ The porosity values are further confirmed by X-CT data,^12^ shown in Fig. S3 (ESI†). Overall, both *τ* and *ε* do not vary appreciably in these materials. The effective diffusion coefficient according to eqn (1) is also essentially identical and, thus, it cannot be used to differentiate the vastly different fuel cell performance of these two MPLs. The noticeable variation between them lies in the characteristic size of the pores, as it emerges from the pore size distribution plots in Fig. 1f, where the VGCF exhibits up to one order of magnitude larger pores than the Li100.

**Fig. 1:**
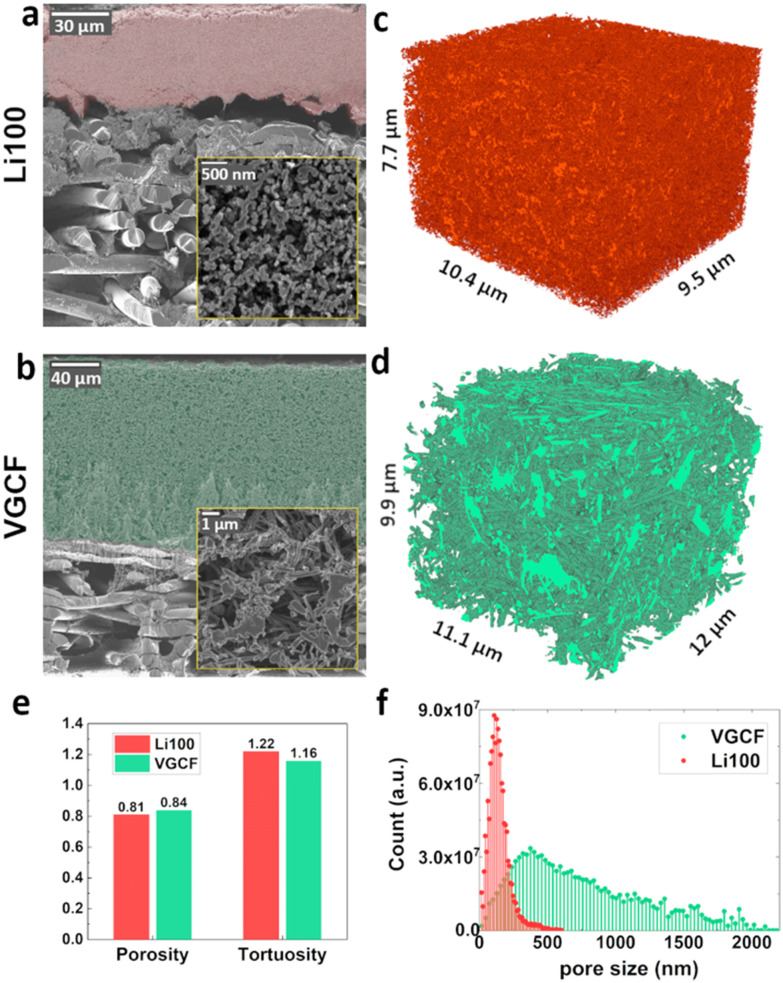
SEM images of Ar^+^ beam polished cross sections of (a) Li100 MPL and (b) VGCF MPL (both coated onto a carbon fibre GDL-S), with the MPL regions shaded in red and green, and insets of each MPL structure. Reconstructed volumes of the Li100 MPL (c) and the VGCF MPL (d) were obtained after embedding. (e) Porosity and tortuosity comparison of the two MPL designs. (f) Li100 and VGCF pore size distribution. Average pore size was 155 nm for Li100 MPL, and 770 nm for VGCF MPL.

To better understand the effect of the pore network on the overall performance of the MPLs, we analyse the volumes on the basis of the connectivity of the network. Fig. 2a and b show the Li100 and VGCF pore network analysis. Starting from the pristine pore volumes with all the considered pore sizes indicated in the legend, we gradually remove pores smaller than the indicated size. At each step, the dark blue region represents the largest connected domain in the network, separated from the light blue disconnected pores. The volume of the connected pores decreases with the increase of the minimum size of the considered pores, revealing the presence of the so-called constrictions of the pores. The process was iterated until it was not possible to find a percolating path of connected pores through the volume, *i.e.* the connected region was not in touch with the two opposite facets of the cube. The loss of percolation occurs at the limit of removing all the pores smaller than *d*_p_ = 230 nm in the case of Li100, while in the case of VGCF this happens at much higher values of *d*_p_ > 1000 nm. The connected pore volume of the VGCF MPL decreases much more slowly than that of the Li100 MPL when the minimum pore size of the network is increased. Therefore, since the constrictions can be defined as narrowing regions of the pore network connecting larger regions, it is expected to find narrower constrictions in Li100 compared to VGCF. This may have noticeable implications for the flow of high surface energy fluids in these porous materials. Other Li100 and VGCF sample volumes have been analysed giving consistent results (Fig. S4, ESI†). Fig. 2c plots the porosity loss as a function of the minimum size of the pores. For each of the two MPL designs, all the analysed sample volumes (labelled as 1, 2, and 3 in Fig. 2c) display the same behaviour. The total pore volume *V*_P_ decreases uniformly with increasing restrictive pore size, according to the pore size distribution. This parameter is not affected by constrictions, since connected and disconnected pores are treated the same way. Fig. 2d shows the loss of percolation by plotting the equivalent percolating volume, which is measured at each minimum size step as the smallest cubic volume containing the connected blue regions of Fig. 2a and b. It is evident that, for both MPL designs, the percolating volume is essentially unaltered from the initial one, until the final step where it drops down to a lower value. The porosity loss suggests that fewer percolating paths are available within the MPL sample volume when the minimum pore size is increased, even before the steep drop in percolation.

To quantify the effect of constrictions on the pore network, a descriptor for the loss of connectivity is introduced. We define the fraction of connected pores 
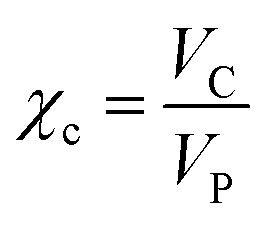
 as the volume ratio between the largest connected region and the total pore volume at each step. Fig. 3 plots *χ*_c_ as a function of the minimum considered pore size for all Li100 and VGCF volumes. Both plots are divided in three regions where *χ*_c_ exhibits distinct behaviour. Upon removal of small pores, the value of *χ*_c_ is close to the one of the native pore network, *i.e.* unity (orange-shaded regions in Fig. 3), since all the pores remain interconnected despite the porosity loss. This region is named constrictions-free pore size region. In the transition region, *χ*_c_ decreases without loss of percolation through the initial volume, while at increased minimum pore size (purple-shaded regions in Fig. 3) no more percolating paths exist and *χ*_c_ reaches its lowest value. By further increasing the minimum pore size, an increase of *χ*_c_ is observed for Li100 in Fig. 3a (and, to a lesser extent also for VGCF in Fig. 3b), which is due to the fact that fewer pore regions remain outside of the largest connected one, thus raising the *V*_C_/*V*_P_ ratio. The extension of the constrictions-free region goes from 0 nm up to the pore size where the connectivity becomes limited. This pore size is defined as the characteristic constrictions size, *d*_C_, and it has noticeably different values for the two MPL designs: *d*_C,Li100_ = 120 nm and *d*_C,VGCF_ = 700 nm. This variation has relevant impact whenever the diffusing species flow through larger size pores, which may happen in the case of high surface energy liquids, or whenever smaller size pores are saturated. Additionally, constrictions may reduce the expected liquid flow through a volume of given porosity and tortuosity.

For PEM fuel cells, the interest in pore network analysis using the minimum size as a parameter comes from the capillary pressure driven liquid water removal from the catalyst layer and the MPL. Constrictions may be responsible for water confinement and the saturation of pores with liquid water, especially in the case of hydrophobic MPLs. The existence of larger volume percolating paths in the VGCF MPL (rather than in the Li100 MPL) would therefore explain its better performance under high relative humidity conditions. To experimentally evaluate the effect of constrictions, mercury intrusion porosimetry (MIP) measurements were performed. Fig. 4a and b display the cumulative intruded volume measured with MIP and FIB/SEM tomography for both MPL designs. The cumulative volume was computed by summing all the voxels of each pore size, starting from the largest pores. The grey-shaded regions of the plot, for each of the two MPLs, are the constrictions-free pore size regions in Fig. 3, which goes from zero up to what has been defined as characteristic constrictions size. The main difference between the porosimetry and the tomography curve is the sudden change of the derivative of the porosimetry curves near the characteristic constrictions size of the MPLs. This is associated with the intrusion mechanism of Hg. The pressure during MIP measurements is inversely proportional to the radius of the pores,^10^ given by the Washburn equation (*i.e.*
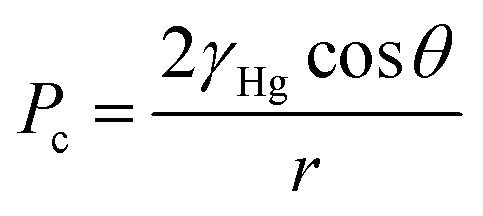
), meaning that the progressive filling of the pores starts from the larger ones. Whenever larger pores are surrounded by smaller size constrictions, they are not directly accessible by Hg intrusion, and the pressure, *P*_c_, to fill them is increased by a factor 
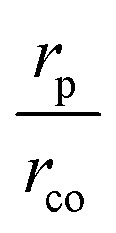
, where *r*_co_ is the constriction equivalent radius and *r*_p_ the large pore equivalent radius. This leads to an overestimation of the filled volume of the pores below the characteristic constrictions size and to an underestimation of the larger pores in the MIP measurements. Therefore, the sudden change in the derivative of the MIP curves is related to the characteristic constrictions size, which is in good agreement to the behavior predicted with FIB/SEM tomography. The linear behavior of the Hg porosimetry curves at large pore sizes is associated with the elastic deformation of the soft and porous structure that can be compressed under Hg pressure. Thus, the better performance of the VGCF MPL under high relative humidity conditions is attributed to the more efficient liquid water removal from the catalyst layer and the MPL since the characteristic constrictions size is higher and larger percolating paths are available through the MPL.

**Fig. 2:**
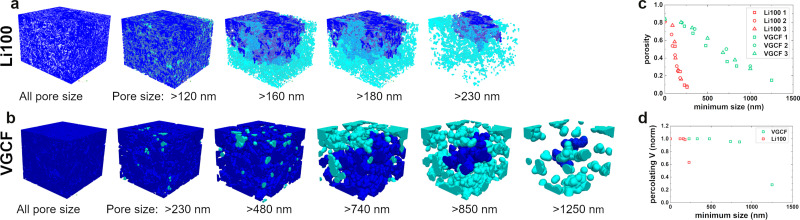
Pore network analysis of MPLs based on (a) Li100 and (b) VGCF, where small size constrictions are progressively removed, leaving only the larger pores. The dark blue region in each volume represents the largest connected domain of pores and the volumes are displayed up to the loss of percolation through each volume. (c) Porosity plot of all Li100 and VGCF volumes measured. The displayed volumes in (a) correspond to volumes 1 of Li100 and VGCF (open square symbols). (d) Percolating volume plot of the displayed in (a) Li100 and VGCF, as a function of the minimum considered pore size.

**Fig. 3:**
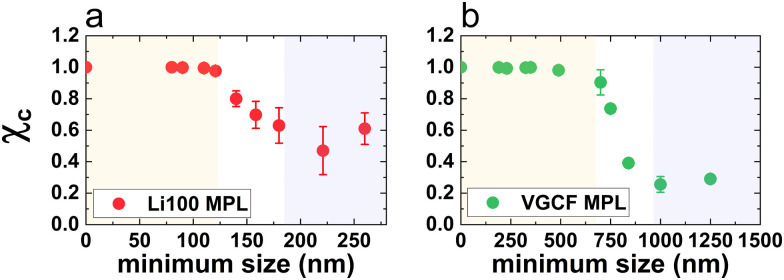
Fraction of connected pores referenced to the total pore volume of the MPL, plotted as a function of the minimum pore size for (a) Li100 and (b) VGCF MPL. Note that each point corresponds to the volumes that the particular minimum size could be extracted.

**Fig. 4:**
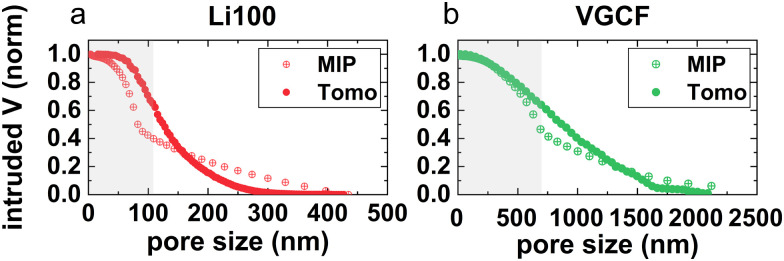
Comparison of the cumulative intruded pore volume obtained by MIP with the available volume for each pore size obtained by FIB–SEM tomography. For both (a) Li100 and (b) VGCF MPLs, the change in the derivative of the porosimetry curve corresponds to the constrictions value. Upper limits of grey shaded areas indicate the constrictions size values obtained with tomography.

To conclude, two different MPL designs have been investigated with FIB/SEM tomography that includes a 3D reconstruction for a VGCF based MPL, which performs better under high-RH conditions. The similarity of porosity and tortuosity values confirmed that mass transport limitation is due to the pore size and the analysis of the 3D volumes revealed the role that the characteristic size of pore constrictions play for determining possible percolating paths of liquid water. A direct comparison between MIP and tomographic data further supports the hypothesis of a key role played by constrictions in pore saturation, by showing this effect on a high surface energy intruding liquid. The characteristic constrictions size is proposed as a descriptor to evaluate water saturation in gas diffusion electrodes and layers and it can be extended to other systems where diffusing species of a given size flow through a sub-μm scale pore network, such as nanoparticles in various filtering systems.

The work was supported by the Swiss National Research Foundation (SNF) under award no. CRSII5_180335.

## Conflicts of interest

There are no conflicts to declare.

## Supplementary Material

CC-058-D2CC02844A-s001
