# Supplementary material for: On the role of pore constrictions in gas diffusion electrodes
Source: Chem Commun (Camb). 2022 Jul 13;58(63):8854–7. doi: 10.1039/d2cc02844a (PMC9350991; doi:10.1039/d2cc02844a)
Supplement: CC-058-D2CC02844A-s001 [file CC-058-D2CC02844A-s001.pdf]

Electronic Supplementary Material (ESI) for ChemComm.
This journal is © The Royal Society of Chemistry 2022

Supporting information for

On the role of pore constrictions in gas diffusion electrodes

Michele Bozzetti,[1] Anne Berger,[2] Robin Girod,[1] Yen-Chun Chen,[3] Felix N. Büchi,[3] Hubert Gasteiger[2] and Vasiliki Tileli[1*]

[1]Institute of Materials, Ecole Polytechnique Federale de Lausanne, Station 12, 1015 Lausanne, Switzerland

[2]Chair of Technical Electrochemistry, Department of Chemistry and Catalysis Research Center, Technische Universität München, Germany

[3]Electrochemistry Laboratory, Paul Scherrer Institute, 5232 Villigen PSI, Switzerland

Corresponding author

*Vasiliki Tileli: vasiliki.tileli@epfl.ch

This file contains: Methods and Supplementary Figures 1-4

Methods

Sample preparation: A detailed description of the MPL fabrication is described in Refs 1 and 2 for the Li100 and VGCF respectively. The evaluated MPLs consist of 20 wt-% PTFE binder (Dispersion: TF 5035GZ from 3 M Dyneon) and 80 wt-% to two different types of carbon: a conventionally used high surface area carbon black, referred to as Li100 (from Denka, Japan; specifications: BET surface area = 68 m^2/g, average particle size = 35 nm), and vapor grown carbon fibers, referred to as VGCF (from Showa Denko; specifications: BET surface area = 13 m^2/g, fiber length = 10–20 µm, fiber diameter = 150 nm). To create an MPL slurry, the carbon powder and PTFE dispersion were mixed with water (Milli-Q, 18MΩ) as a solvent, methyl cellulose (Sigma-Aldrich) as thickener, and Triton X-100 (Sigma Aldrich) as an emulsifier. The slurry was coated on a Freudenberg H14 (PTFE treated) gas diffusion layer substrate (GDL-S) using a doctor blade and a stainless steel stencil. For mercury intrusion analysis, the MPL slurry was coated on a glass plate from where it could be removed as a freestanding MPL layer. The final MPL/GDL-S combinations or the freestanding MPLs were subject to a heat-treatment procedure as described in reference[1].

Embedding of both samples was accomplished by atomic layer deposition (ALD) of zinc oxide with a BENEQ TFS200 instrument. Diethyl zinc (DEZ) and H_2O were used as precursors, the chamber temperature was set at 200°C, while the precursors were not pre-heated. Refractometer characterization revealed that a ZnO layer of ~ 100 nm was deposited on the sample surface, with the layer thickness decreasing by going into the sample. A penetration depth of 20 µm was reached. The deposition consisted of 1000 cycles, with pulse duration of 600 ms for both H_2O and DEZ, with 900 ms waiting time after each pulse, and a purge time of 3 s. Ilion II by Gatan was used for the argon beam surface polishing, using a 4 keV argon beam in dual beam cross section polishing

mode.

FIB/SEM microscopy: ZEISS CrossBeam 540 FIB-SEM equipped with Atlas 5 tomography software was used for volume acquisition. Milling conditions for the focused ion beam were 30 keV and 700 pA, while scanning electron beam parameters were 1.5 keV and 500 pA. The Voxel size was 8 nm for Li100 MPL and 10 nm for VGCF MPL. Tilt correction by Atlas 5 was applied to obtain isotropic voxels.

Image processing: Volume reconstruction and segmentation for Li100 was entirely done with Fiji, using the WEKA machine learning tool included in ImageJ[3]. For the VGCF MPL, stack registration and volume reconstruction were performed with Fiji and the alignment of the slices was performed using three FIB-milled notches on the top surface of the sample. A pixel classifier based on deep learning, YAPiC Python toolbox, was used to segment the fibers from the pores. One in a hundred z-sections was selected for the training, that consisted of manual annotations using the QuPath software for ground truth data[4], and a U-Net (2D) architecture[5], with minibatch-wise normalization, data augmentation by rotating and flipping, 20% validation and 50 training steps per epochs. For both the MPL, *local thickness* plugin included in Bonej library[6,7] was used to extract the pore size distribution, while flow-based tortuosity was obtained with TauFactor MATLAB application[8].

Hand thresholding was performed several times on the thickness distribution of each volume, and threshold values were chosen in order to have enough points distributed between the unthresholded pore network and the loss of percolation value. Once the hand thresholding was performed on both volumes to cut off the constrictions, *find connected regions* ImageJ plugin was then used to extract the largest connected pores domain at each step. The plots in Fig. 3 are built up from the volumes displayed in Fig. 2a-b as well as the volumes displayed in Fig. S4. Unlike Fig. 2c, that shows each volume data separately, datapoints of Fig. 3 were obtained by a weighted average among the

different volumes, using the volume size as weight.

Mercury Intrusion Porosimetry: Measurements were carried out on the Autopore Instrument (9600, Micromeritics Instrument Corporation, USA). Freestanding MPL samples (~80-120mg) were cut to small pieces (1cm x 2.5 cm) and inserted in the penetrometer (sample holder) comprised of 5 mL head and 0.392 mL stem volume, which resulted to a stem usage of 60-75%. The pressure range from vacuum to 45 psia was measured in horizontal penetrometer positioning, while the range from 45-61000 psia was measured in the high pressure port in vertical penetrometer position. The pressure was increased to achieve a spacing of 25 points per decade on a logarithmic scale. The pressure p was converted to a pore diameter d_{pore} via Washburn's equation:

$$d_{pore} = -\frac{4 \cdot \gamma_{Hg} \cos\theta}{p}$$

with the surface tension of mercury γ_{Hg} (0.480 N/m) and the contact angle θ (140°). To compare the cumulative intrusion with the tomography results, pores larger than 524 nm and 2119 nm were omitted for the Li100 and VGCF MPL, respectively.

References

1. Simon, C.; Kartouzian, D.; Müller, D.; Wilhelm, F.; Gasteiger, H. A. Impact of Microporous Layer Pore Properties on Liquid Water Transport in PEM Fuel Cells: Carbon Black Type and Perforation. *Journal of The Electrochemical Society* **2017**, *164* (14), F1697–F1711. https://doi.org/10.1149/2.1321714jes
2. Simon, C.; Endres, J.; Nefzger-Loders, B.; Wilhelm, F.; Gasteiger, H. A. Interaction of Pore Size and Hydrophobicity/Hydrophilicity for Improved Oxygen and Water Transport through Microporous Layers. *Journal of The Electrochemical Society* **2019**, *166* (13), F1022–F1035. https://doi.org/10.1149/2.1111913jes
3. Arganda-Carreras, I.; Kaynig, V.; Rueden, C.; Eliceiri, K. W.; Schindelin, J.; Cardona, A.; Sebastian Seung, H. Trainable Weka Segmentation: A Machine Learning Tool for Microscopy Pixel Classification. *Bioinformatics* **2017**, *33* (15), 2424–2426. https://doi.org/10.1093/bioinformatics/btx180

4. (29) Bankhead, P.; Loughrey, M. B.; Fernández, J. A.; Dombrowski, Y.; McArt, D. G.; Dunne, P. D.; McQuaid, S.; Gray, R. T.; Murray, L. J.; Coleman, H. G.; James, J. A.; Salto-Tellez, M.; Hamilton, P. W. QuPath: Open Source Software for Digital Pathology Image Analysis. *Sci Rep* **2017**, *7* (1), 16878. https://doi.org/10.1038/s41598-017-17204-5
5. (30) Ronneberger, O.; Fischer, P.; Brox, T. U-Net: Convolutional Networks for Biomedical Image Segmentation. *arXiv:1505.04597 [cs]* **2015**
6. (31) Doube, M.; Kłosowski, M. M.; Arganda-Carreras, I.; Cordelières, F. P.; Dougherty, R. P.; Jackson, J. S.; Schmid, B.; Hutchinson, J. R.; Shefelbine, S. J. BoneJ: Free and Extensible Bone Image Analysis in ImageJ. *Bone* **2010**, *47* (6), 1076–1079. https://doi.org/10.1016/j.bone.2010.08.023
7. (32) Dougherty, R.; Kunzelmann, K.-H. Computing Local Thickness of 3D Structures with ImageJ. *MAM* **2007**, *13* (S02). https://doi.org/10.1017/S1431927607074430
8. Cooper, S. J.; Bertei, A.; Shearing, P. R.; Kilner, J. A.; Brandon, N. P. TauFactor: An Open-Source Application for Calculating Tortuosity Factors from Tomographic Data. *SoftwareX* **2016**, *5*, 203–210. https://doi.org/10.1016/j.softx.2016.09.002

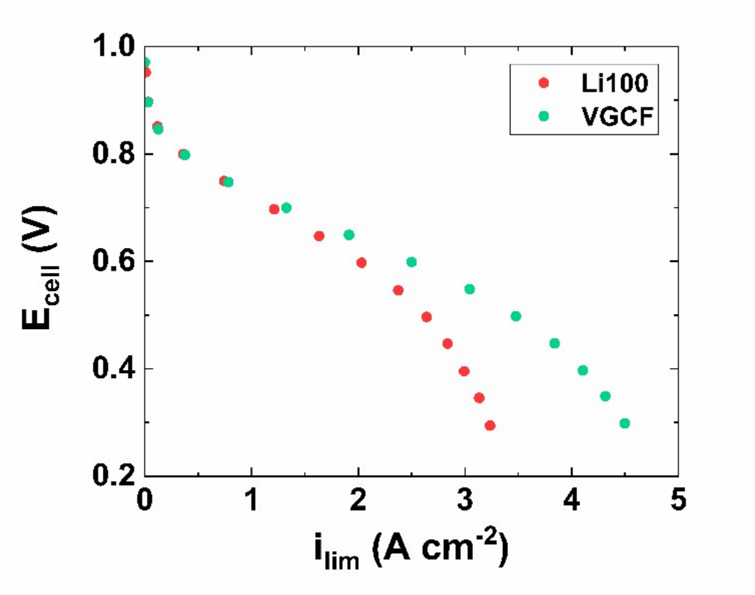

Figure S1: H_2/air performance of the L100 and the VGCF MPLs obtained in a 5 cm^2 single-cell PEM fuel cell under high-RH operating conditions (differential H_2/air flows (2000/5000 nccm) at a cell temperature of 50 °C, a relative humidity of 120 %, and an inlet pressure of 300 kPa_{abs}; obtained with a Gore MEA with an 18 µm membrane and anode/cathode loadings of 0.1/0.4 mg_{Pt}/cm^2). The here shown data were taken from Simon *et al.*[1] for the Li100 data, and from Simon *et al.*[2] for the VGCF data. For both the samples, the MPL thickness was 30 µm coated onto the same GDL-S.

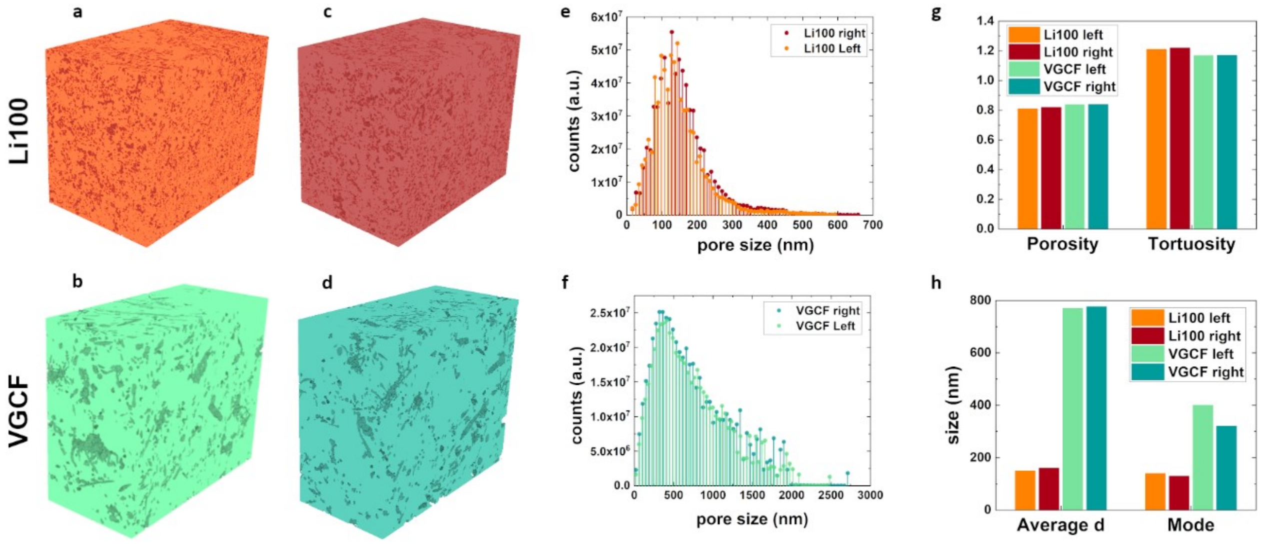

Figure S2: a) Li100 left half of the volume analysed in the manuscript and b) VGCF left half of the volume analysed in the manuscript, corresponding right halves are c) and d). Pore size distributions of the two e) Li100 halves and f) VGCF halves show good consistency, as well as g) porosity and tortuosity values. Looking at size parameters, h) the average pore size for Li100 sub-volumes are 150 nm and 160 nm, while for VGCF they are 770 nm and 780 nm, while the mode values are 140 nm and 130 nm for the Li100 stacks, 400 nm and 320 nm for the VGCF stacks.

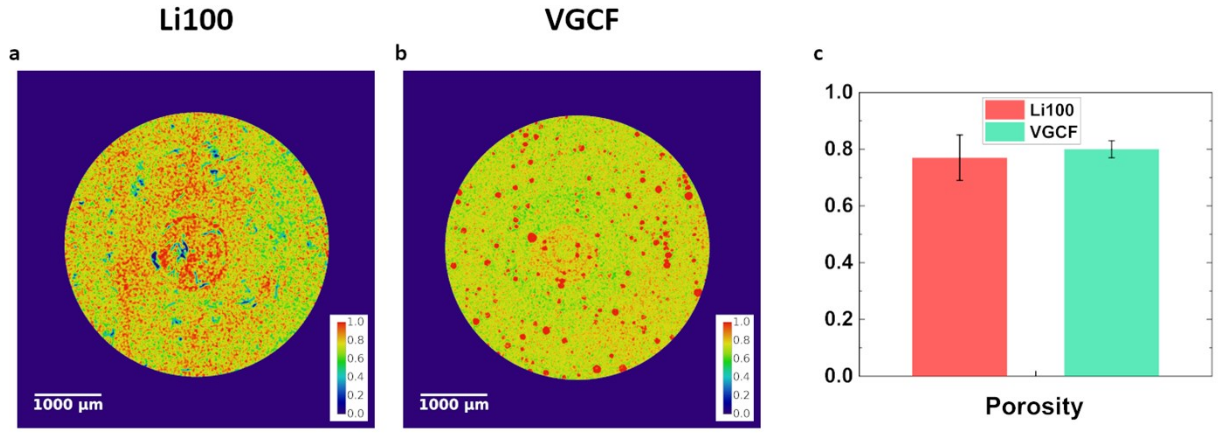

Figure S3: X-CT scans of a) Li100 MPL and b) VGCF MPL. The VGCF MPL porosity value is more affected by compression and therefore only the central (uncompressed) region was taken into account. c) Porosity values of 0.77 for Li100 and 0.80 for VGCF with relative uncertainties of 0.08 and 0.03 were evaluated.

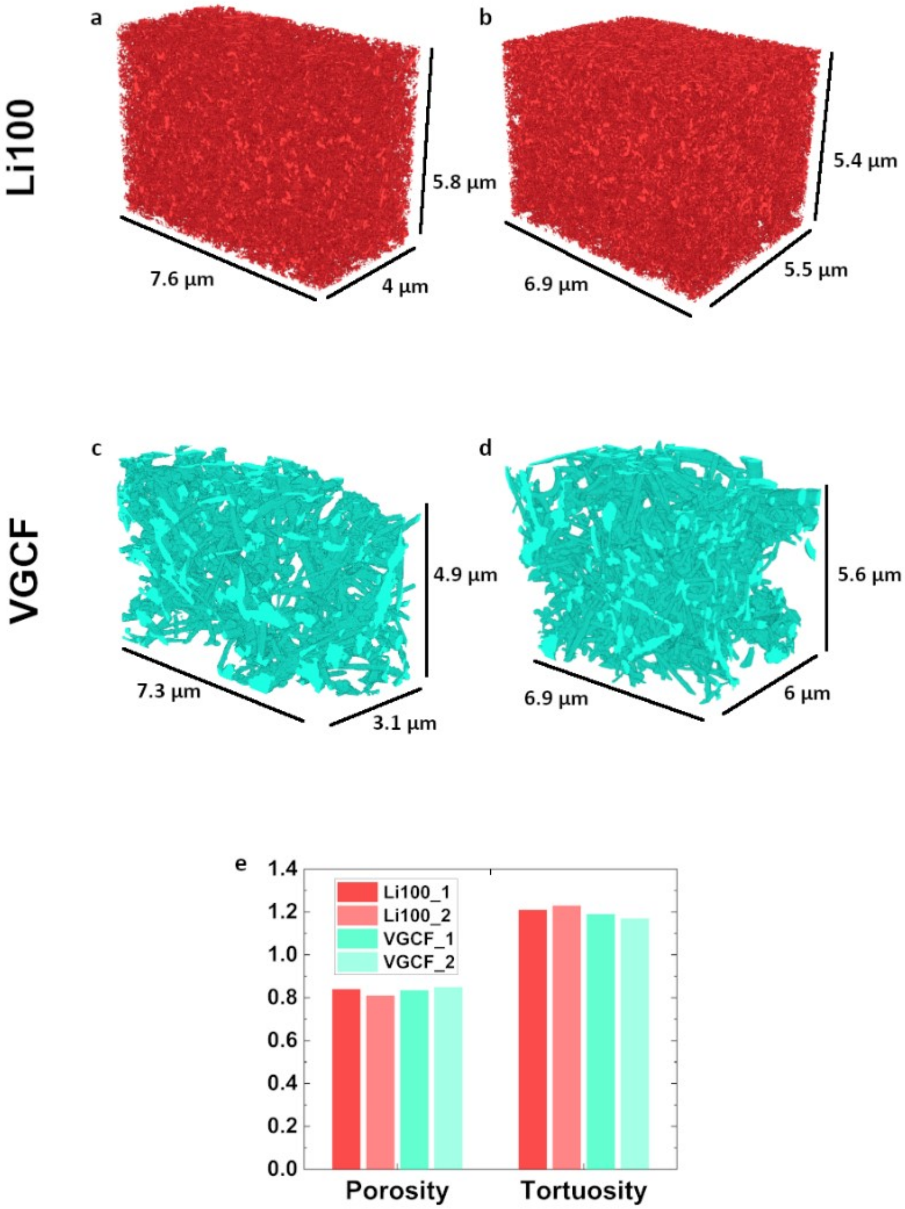

Figure S4: FIB-SEM reconstructed volumes of Li100 MPLs for sample volumes 1 (a) and 2 (b), and of VGCF MPLs for sample volumes 1 (c) and 2 (d). e) Porosity and tortuosity plot illustrating the consistency with the volumes shown in the main manuscript.
